# Tunability of the bandgap of SnS by variation of the cell volume by alloying with A.E. elements

**DOI:** 10.1038/s41598-022-11074-2

**Published:** 2022-05-06

**Authors:** Fumio Kawamura, Yelim Song, Hidenobu Murata, Hitoshi Tampo, Takehiko Nagai, Takashi Koida, Masataka Imura, Naoomi Yamada

**Affiliations:** 1 Research Center for Functional Materials, High Pressure Group, National Institute for Materials Science (NIMS), 1-1 Namiki, Tsukuba, Ibaraki 305-0044 Japan; 2 Department of Materials Science, Osaka Prefecture University, 1-1 Gakuencho, Naka-ku, Sakai, Osaka 599-8531 Japan; 3 National Institute of Advanced Industrial Science and Technology (AIST), Umezono 1-1-1, Tsukuba, Ibaraki 305-8568 Japan; 4 Next-Generation Semiconductor Group, National Institute for Materials Science (NIMS), Namiki 1-1, Tsukuba, Ibaraki 305-0044 Japan; 5 Department of Applied Chemistry, Chubu University, 1200 Matsumoto, Kasugai, Aichi 487-8501 Japan

**Keywords:** Materials science, Solar energy

## Abstract

We clarified that the bandgap of inorganic materials is strongly correlated with their effective coordination number (ECoN) via first-principles calculations and experimental confirmations. Tin mono-sulphide (*Pnma*) and germanium mono-sulphide (*Pnma*) were selected as model cases since these materials successively alter the ECoN as the cell volume changes and show an uncommon relationship between cell volume and bandgap. Contrary to the common semiconductors, the bandgaps of SnS *(Pnma)* and GeS *(Pnma)* have a positive relationship with respect to cell volume. This unique phenomenon was explained by incorporating the concept of ECoN into the theoretical studies. The theory proposed in this study is widely applicable to semiconductors with low-symmetry structures. Further, we experimentally demonstrated that the bandgap of SnS *(Pnma)* can be broadly tuned by changing the unit cell volume via alloying with alkali-earth (A.E.) metals, which could allow SnS to be applied to Si-based tandem photovoltaics. Alloying with A.E. elements also stabilised Cl as an *n*-type donor, which enabled *n*-type conduction in the bandgap-widened SnS film in the SnS-based semiconductors.

## Introduction

For applications, SnS (*Pnma*) may be useful in next-generation solar cells and thermoelectric materials^1–3^ since it is composed of non-toxic and earth-abundant elements, similar to Cu_2_ZnSn(S_*x*_,Se_1−*x*_)_4_^4–7^. Mono-sulphide SnS films have been fabricated by methods including sputtering^8–12^, atomic layer deposition^13–16^, chemical vapour deposition^17–20^, pulsed laser deposition^21^, chemical bath deposition^22–25^, spray pyrolysis^26,27^, spin coating^28^, physical vapour deposition^29–32^, and molecular beam epitaxy^33^. Noguchi et al. proved that SnS (*Pnma*) can function as a photovoltaic absorber^34^, and SnS (*Pnma*) solar cells have recently been developed owing to the establishment of *n*-type doping.

Sn–S semiconductors have several structures and compositions (SnS (*Pnma*, *Cmcm*, *Fm*-3* m*, *P*2_1_3, and *F*-43* m*), Sn_2_S_3_, and SnS_2_))^35,36^. Chattopadhyay et al. investigated the stability of the mono-sulphide (SnS) using neutron diffraction and revealed that SnS (*Pnma*) transforms into SnS (*Cmcm*) via a soft-phonon mode^37^. Skelton et al. investigated the stability of the Sn–S system using first-principles calculations and showed that SnS (*Pnma*) has the highest stability, although the stabilities of SnS (*Pnma*) and SnS (*Cmcm*) are quite close^38^. Due to the similar stabilities of Sn–S compounds, fabricating single-phase SnS (*Pnma*) films remains difficult. Furthermore, the bandgaps of Si and SnS (*Pnma*) are close, making it difficult to use SnS as the top cell in Si-based tandem solar cells. Therefore, it is necessary to widen the bandgap of SnS towards *E*_g_ ~ 2 eV. The difficulty of fabricating *n*-type SnS (*Pnma*) films with reasonable electrical properties is another drawback of SnS-based devices, although *n*-type SnS as sintered compacts and single crystals has been prepared by flux growth^39^.

In this study, tuning the bandgap of SnS towards a suitable range for the top cell of a Si-tandem solar cell was achieved by alloying A.E. elements to increase the cell volume based on results from the first-principles study. We emphasize that increasing the cell volume has not been confirmed to cause bandgap widening. The tendency for the bandgap of compound semiconductors to widen as the unit cell volume decreases has been broadly applied to tune the bandgaps of materials such as GaN-based semiconductors^40,41^. The uncommon bandgap behaviour observed in SnS (*Pnma*) could be due to the effect of the coordination number (C.N.) on the bandgap value.

In addition, the SnS alloyed with A.E. elements also showed chlorine trapped in the crystals, which enabled us to determine an n-type conduction.

## Results

### Theoretical prediction of SnS (*Pnma*) and GeS (*Pnma*) bandgaps

**Figure 1:**
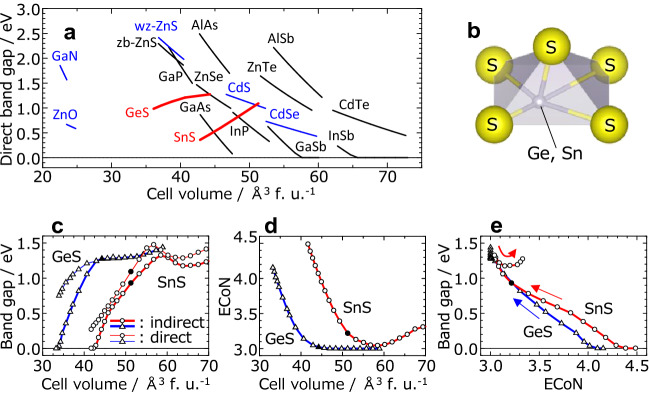
Variation in the bandgap of typical semiconductors and the uncommon behaviour of SnS and GeS. (**a**) Volume dependence of direct bandgap in compound semiconductor materials calculated with GGA-PBE between 0 and 8 GPa. Black and blue lines represent zinc-blende and wurtzite structures, respectively. (**b**) Coordination structure of cations in *Pnma*-type SnS and GeS. Relationships between (**c**) unit cell volume and bandgap (thin and thick lines denote indirect and direct bandgaps, respectively), (**d**) unit cell volume and effective coordination number (ECoN), and (**e**) ECoN and bandgap of SnS and GeS. Closed marks indicate the values at 0 GPa.

The uncommon bandgap behaviour with respect to unit cell volume of SnS and GeS calculated in this study is depicted in Fig. 1a. The bandgaps of SnS and GeS increase with unit cell volume, which is opposite to the tendency of common semiconductors like wurtzite-based and zinc-blend-based materials. A 20% expansion in unit cell volume causes the bandgap of SnS to widen by 0.7 eV as indicated in Fig. 1a. Band structure of SnS with different unit cell volume is given in the supplementary Fig. S1. To elucidate this behaviour, it was analysed from a structural viewpoint. SnS and GeS have lower-symmetry structures than common semiconductors, as shown in Fig. 1b, and 3 + 2 coordination environments around the cations in the ground state (0 GPa). With changes in unit cell volumes, coordination environments also change as shown in supplementary Fig. S2. The effective coordination number (ECoN) of SnS and GeS changes significantly (as much as 1.5) upon expansion or shrinkage of the unit cell in the examined pressure range. The relationships between the bandgap, unit cell volume, and ECoN are summarised in Fig. 1c–e. Figure 1c shows that both the indirect and direct bandgaps decrease with the unit cell volume at small volumes. In contrast, the bandgaps do not monotonically change above *E*_g _≈ 1.3 eV. Figure 1d shows the massive change in ECoN induced by varying the unit cell volume; the shape of Fig. 1d is a nearly vertical mirror image of that in Fig. 1c. This naturally implies the relationship between bandgap and ECoN illustrated in Fig. 1e, indicating that the bandgaps of SnS (*Pnma*) and GeS (*Pnma*) are dominated by the ECoN. The unusual bandgap behaviour of SnS (*Pnma*) and GeS (*Pnma*) can thus be interpreted as arising from a significant change in ECoN. According to our findings, we state that the bandgap is strongly negatively correlated with the ECoN. We note that this effect is not specific to SnS and GeS but is applicable to all inorganic materials.

### Fabrication of bandgap-widened SnS films

**Figure 2:**
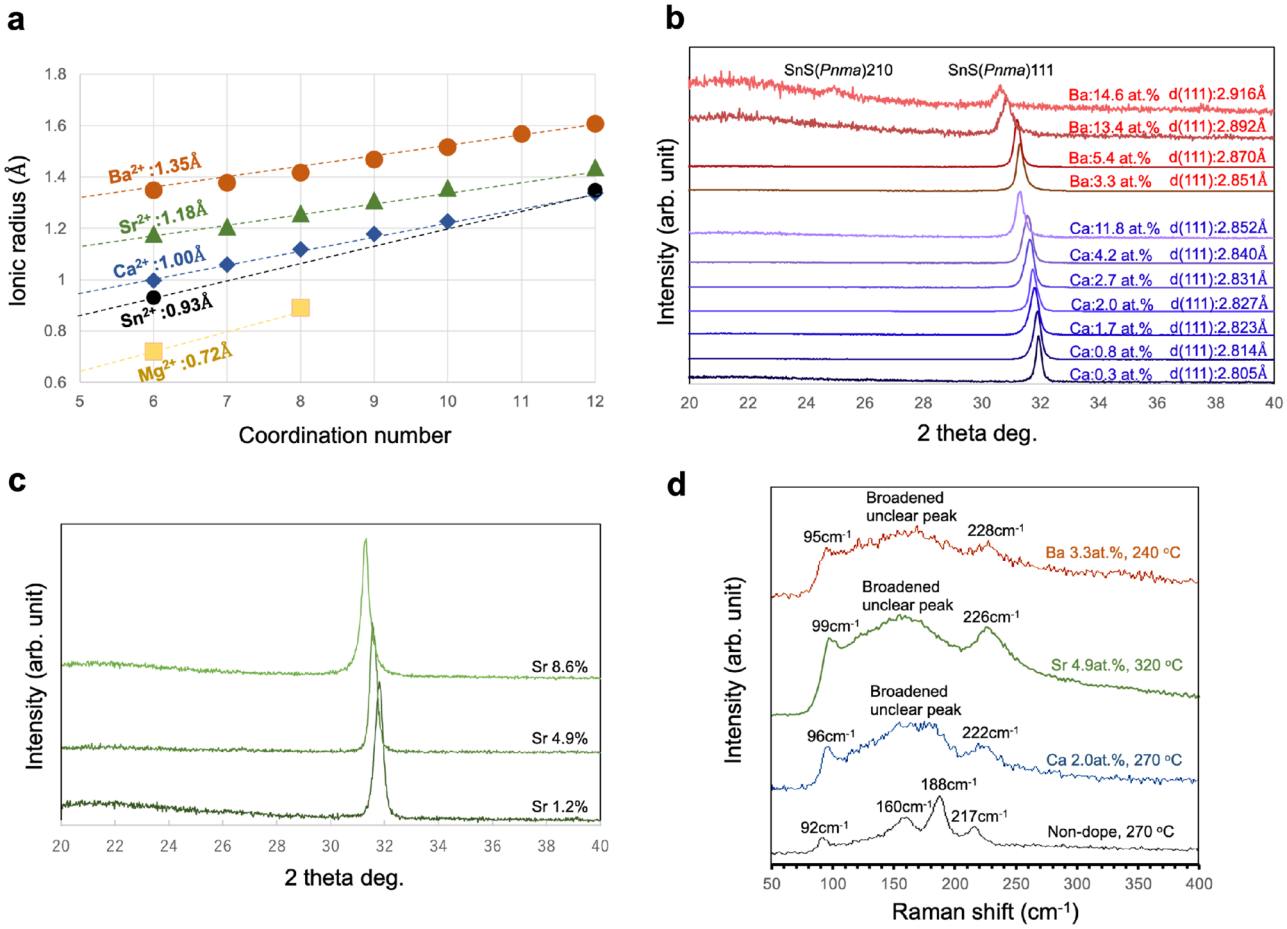
Change in unit cell volume by alloying the SnS film with A.E. elements and confirmation of structure. (**a**) Dependence of the ionic radii of A.E. elements on the C.N. Ba^2+^, Sr^2+^, and Ca^2+^ are candidates for expanding the unit cell volume of SnS by replacing the Sn site. (**b**, **c**) Dependence of the XRD peak position of SnS (*Pnma:* JCPDS card number 23-0677 on the amount of alloyed A.E. element (Ba^2+^, Sr^2+^, and Ca^2+^). The 111 peak continuously shifted towards lower angles with increasing A.E. element content. No peaks aside from 111 were observed except for the highly Ba-alloyed SnS film. (**d**) Raman spectra of Ba-, Sr-, and Ca-alloyed and pure SnS films. Neither film contained any Sn–S phases other than SnS (*Pnma*).

Based on the theoretical study described above, we alloyed SnS with Ca, Sr, and Ba using RF-magnetron sputtering to expand the unit cell. The ionic radii of these alkaline-earth (A.E.) elements are compared in Fig. 2a. To expand the unit cell, A.E. elements are more suitable than other divalent cations since of the non-A.E. elements, Pb^2+^ is the only divalent cation with a larger ionic radius than^42^ Sn^2+^. Fig. 2 also shows the concrete values of the hexacoordinated state of each element. Among the A.E. elements, Ca, Sr, and Ba are promising for replacing the Sn site in SnS to expand the unit cell.

The amount of alloyed A.E. elements in the SnS films was controlled by adjusting the RF power applied to each target. The XRD profiles of Ca-, Ba-, and Sr- alloyed SnS are shown in Fig. 2b,c. The SnS 111 peak gradually shifted towards lower angles with increasing A.E. content, proving that the cell volume could be increased by alloying with A.E. elements having larger ionic radii than Sn^2+^. Interestingly, the XRD patterns contained only the 111 and 222 peaks. No peaks other than {111} were observed even with a log-scale vertical axis, except for the 210 peak for the film with maximum Ba-alloying of 14.6 at.%, which indicated a strong preferential <111> orientation. Over-alloying may result in deterioration of crystallinity and weakened orientation. Too little information was obtained from the XRD measurements to determine the crystal structures. Therefore, the Raman spectra of the pure SnS and Ba-, Sr-, Ca-alloyed SnS films were measured to determine the deposited Sn–S phase.

Figure 2d shows the Raman spectra of the pure SnS and Ba-, Sr-, Ca-alloyed SnS films. All the peaks were assigned to SnS (*Pnma*), and the representative Sn_2_S_3_ and SnS_2_ peaks at 250 and 313 cm^−1^, respectively^11,43^, were absent, which proved that all the films were single-phase SnS (*Pnma*). The measured X-ray Photoelectron Spectroscopy (XPS) data for Ba-alloyed SnS film was indicated in supplementary Fig. S3. We confirmed that an extremely high H_2_S/Ar ratio for the flowing gas generates a Sn_2_S_3_ phase, although the data were not included in the paper. We used an H_2_S/Ar ratio of 0.2, which formed an SnS (*Pnma*) single phase throughout the experiment. We observed whether the phase of the deposited film was SnS, Sn_2_S_3_, SnS_2_, or a mixed phase in each deposition.

Alloying with A.E. elements broadened the peak, which implies that A.E. elements degrade the crystallinity, while all peaks appearing in the alloyed films were assigned as *Pnma*-type.

There have been no reports of preferred <111> oriented single-phase SnS (*Pnma*) films grown by sputtering. The oriented film obtained here may be due to the use of H_2_S gas as the sulphur source. We presume that the H_2_S gas easily decomposes during sputtering, resulting in strongly oriented films without cogenerating any other Sn–S phases.

**Figure 3:**
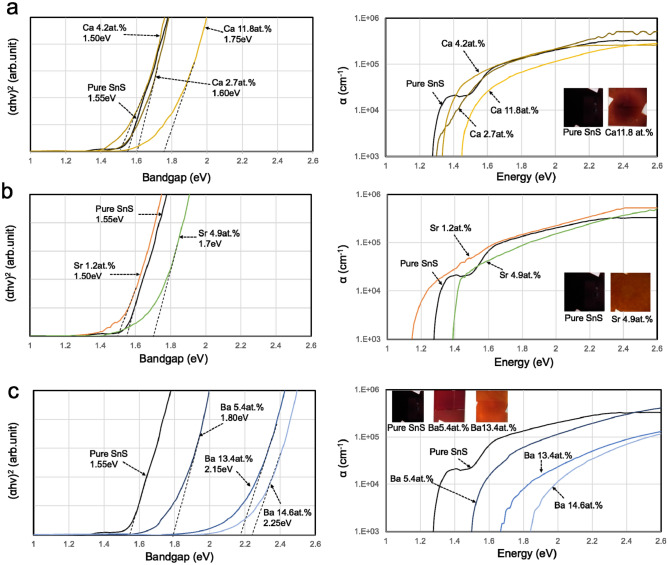
Optical bandgaps estimated by Tauc plots of (*αhν*)^2^ (left) and absorption coefficients (right). Results for the (**a**) Ca-, (**b**) Sr-, and (**c**) Ba-alloyed SnS films. Alloying with Ba, which has the largest ionic radius of the A.E. elements except Ra, resulted in a bandgap greater than 2 eV. The insets in the graph show photographs of the films fabricated with and without A.E. element alloying.

The change in the bandgap upon alloying with Ca, Sr, and Ba was investigated using spectrophotometry. The Tauc plots and absorption coefficients (α) of the Ca-, Sr-, and Ba-alloyed SnS films are shown in Fig. 3a–c, respectively. The results showed that alloying with A.E. elements having larger ionic radii than Sn^2+^ enlarged the bandgap of SnS, as predicted by the theoretical calculations. The SnS thin film fabricated by RF-magnetron sputtering with a gaseous H_2_S source showed a sharp decrease in α in the low-energy region, implying an excellent crystallinity. The measured α was beyond the order of 10^5^ cm^−1^ even for the films with bandgaps above *E*_g _≈ 2 eV.

The same effect has been reported in Ag-doped SnS films, although the authors did not mention the relationship between the unit cell volume and bandgap^26^. With increasing amount of Ag dopant, the optical bandgap increased in the SnS films synthesised by spray pyrolysis. The same group also reported that doping SnS with Al decreased the bandgap, which can be regarded as the same effect.

Although determination of the absolute value of the bandgap from the Tauc plot is a controversial issue, as Oba et al. described in Ref^44^, expansion of the bandgap is proven to be effective in the creation of SnS tandem solar cells.

In contrast, Raadik et al. found that coexistence of small amounts of cubic SnS phase increases the bandgap of SnS (*Pnma*), meaning that the precise bandgap should be examined using advanced methodologies such as photoreflectance and electro-reflectance methods^43,45^.

In the future, we will examine this precise bandgap and the possibility of coexistence of another SnS phase.

### *p*- to *n*-type conversion of SnS films

**Figure 4:**
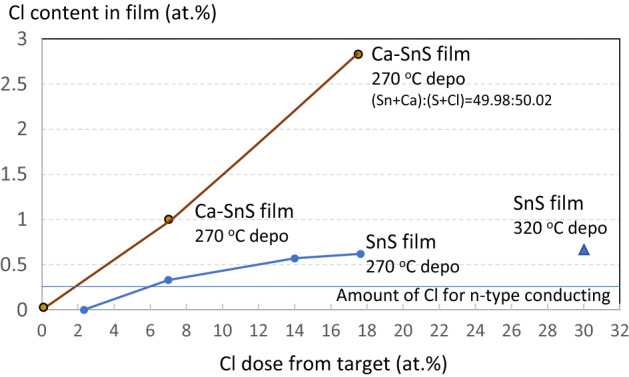
Cl content in as-deposited SnS film and Ca-alloyed SnS film.

Although an *n*-type SnS film induced by Pb doping was reported in 2015^46^, no further progress has been achieved. Cl doping of SnS (*Pnma*) was established by Yanagi et al.^39^, and then the growth method of *n*-type SnS single crystals which could be used for the single crystal substrate was developed. Recently, homojunctions of SnS using *n*-type single-crystal substrates were realised^47^. In this study, Cl doping of the Ca-alloyed SnS films was attempted. We experimentally investigated the origin of the expanded bandgap using ultraviolet photoelectron spectroscopy, X-ray photoelectron spectroscopy, and inverse photoelectron spectroscopy and found that it is caused by the lowering of the valence band maximum; the details will be published in another paper. A lower valence band maximum would be preferable for realising *n*-type conduction. Cl doping was attempted using an SnCl_2_-mixed-Sn target for RF-magnetron sputtering, and the Cl doping amount in the Ca-alloyed SnS films is plotted in Fig. 4.

Figure 4 shows the amount of Cl taken into the Ca-alloyed SnS films. The content of Cl in the film was measured using energy dispersive x-ray spectroscopy (EDX) equipped with scanning electron microscopy (SEM). The amount of Ca was adjusted to approximately 2 at.%, although a slight deviation was observed between samples due to parameters such as the deposition temperature, deposition rate, sputtering effectiveness of each target, etc. The results showed that Ca alloying increased the amount of Cl taken into the film compared with samples without doping; the same effect was also observed with the Sr- and Ba-alloyed films. The straight blue line in Fig. 4 indicates the amount of Cl required to realise *n*-type conduction according to Iguchi et al.^44^. The amount of Cl in the A.E.-alloyed SnS films easily reached this threshold. Further, rapid thermal annealing at 580 °C for 10 s proved that Ca in the film stabilised the Cl contained in the film. After thermal annealing, none of the Cl in the Ca-alloyed SnS film had escaped, whereas the Cl in the pure SnS film was completely removed. Iguchi et al. reported that Cl doped into SnS tends to segregate^48^, suggesting that the Cl dopant in pure SnS has a low stability, like indium in InGaN semiconductors. The stabilising effect of A.E. elements on Cl is promising for realising *n*-type conduction. Although the Cl content taken into the Ca-alloyed SnS film was markedly above the threshold for *n*-type conduction, Hall effect measurements revealed that all the films exhibited *p*-type conduction. We believe this may be attributable to the generation of both *V*_Sn_ and *V*_S_.

To realise *n*-type conduction in A.E.-alloyed SnS, a reaction with argon-diluted HCl gas at 100 °C was attempted in a quartz tube. Unfortunately, the film decomposed within a few minutes of exposure to HCl gas, implying that the formation of SnCl_4_ on the surface of the film is sure to be the origin of the decomposition of the film. These results inspired a different strategy of doping Cl element while suppressing the decomposition of Ca-alloyed SnS. A novel, easy doping technique using SnCl_4_ was developed as described in the next section.

### Development of Cl doping technique for conversion from *p*-type to *n*-type conduction

The conversion of p- into n-type in SnS semiconductor using SnCl_2_ was confirmed by Spalatu et al.^49^ They reported that the carrier type conversion was caused by the phenomenon that SnCl_2_ worked as flux for the regrowth of SnS.

Here we tried to use the liquidus SnCl_4_ in order to supply Cl element into SnS for the first time. The SnCl_4_ is a hopeful alternative to SnCl_2_ because the liquidus SnCl_4_ was expected to diffuse rapidly and cover the film homogeneously on the SnS at low temperatures.

**Figure 5:**
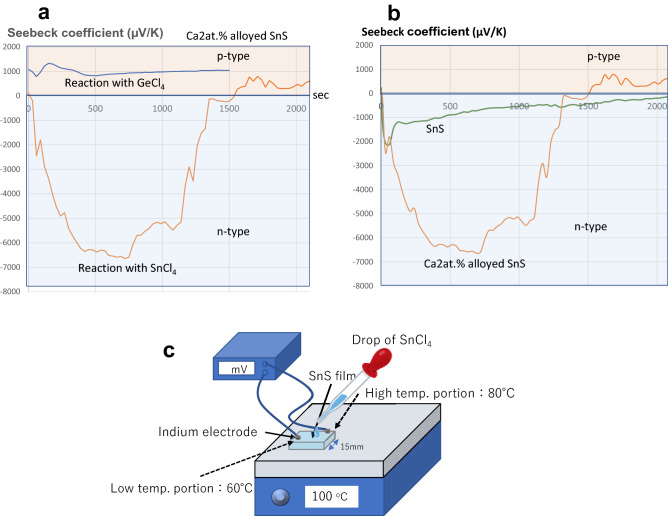
Time-dependent Seebeck effect measurement. (**a**) Results of time-dependent Seebeck effect measurements after liquid SnCl_4_ or GeCl_4_ were dropped on the Ca 2 at.% alloyed SnS film. (**b**) Comparison between alloyed Ca 2 at.% and pure SnS films in terms of time-dependent Seebeck effect measurements. (**c**) Schematic for the time-dependent Seebeck effect measurements.

We used an apparatus that can measure the variation in the Seebeck coefficient (*S*) with time. Liquid SnCl_4_ or GeCl_4_ was dropped on the heated Ca-alloyed SnS film and pure SnS film during continuous *S* measurement. The detailed measurement process is described in the Methods section. Figure 5a–c shows the time-dependent change in *S* after dropping liquid SnCl_4_ or GeCl_4_ on the Ca 2 at.% alloyed SnS and pure SnS films with a temperature gradient from 60 and 80 °C for the low- and high-temperature regions, respectively. The temperature difference of 20 °C caused by the different positions on the hot plate is enough to precisely measure *S*. When SnCl_4_ was dropped on the films, *S* rapidly changed from positive to negative, indicating *n*-type conduction. Dropping SnCl_4_ on the Ca-alloyed SnS film yielded a significantly negative *S* of − 6500 (µV/K), which gradually became less negative with time. The *n*-type conduction changed to *p*-type 1600 s after dropping SnCl_4_. The continuous change in *S* made it impossible to perform Hall effect measurements. Tin chalcogenides have been researched as thermoelectric materials because of their large *S* values. For SnSe, a large *S* of + 7863 (µV/K) at 42 K was reported by Urmila et al., which supports our data^50^. The return from *n*- to *p*-type conduction was surely due to the film reacting with the humidity in the surrounding atmosphere because intentional spraying moist air onto the *n*-type film immediately converted the negative voltage to a positive one.

To investigate this *p*- to *n*-type conversion, the same measurement was carried out using liquid GeCl_4_ instead of SnCl_4_. Interestingly, GeCl_4_ did not cause the conversion from *p*- to *n*-type. The minute change in *S* at the beginning of the measurement, as shown in Fig. 5, was probably caused by the change in electrode temperature upon dropping the cold liquid GeCl_4_ onto the sample. The clear difference between dropping SnCl_4_ and GeCl_4_ implied that the conversion from *p*- to *n*-type conduction is not attributable only to a reaction with Cl. After the experiment, the Cl content was measured to check if Cl had replaced the S sites in the film. The amount of Cl in both the SnCl_4_- and GeCl_4_-treated films was at least 5 at.%, clearly indicating that some of the sulphur in the Ca-alloyed SnS film was replaced with Cl. Figure 5b compares the pure SnS and Ca-alloyed SnS films for this experiment. Although the conversion from *p*- to *n*-type also occurred with the pure SnS sample, the corresponding *S* was markedly smaller. The larger *S* for the Ca-alloyed film indicated that Ca taken into the film might contribute to the observed conversion. Additionally, the lack of *p*- to *n*-type conversion using GeCl_4_ implied that the phenomenon is not only due to Cl doping but also compensation of *V*_Sn_. Although the detailed mechanism explaining the difference between the results for SnCl_4_ and GeCl_4_ has not been specified thus far, SnCl_4_ may not only supply Cl as the donor but also compensate the Sn-vacancy even at significantly low temperatures, which enhances the conversion from p- to n-type. However, GeCl_4_ was probably not able to compensate for the Sn-vacancy due to the difference in ionic radius. The phenomenon that liquid halide converts the conduction type from p- to n-type may be mainly due to compensation for the Sn-vacancy, although doping of Cl is evidently necessary. We plan to investigate this further in future work.

In summary, a strong correlation between the bandgap and C.N. of inorganic materials was demonstrated via first-principles calculations and experiments using SnS (*Pnma*) as a model. A strange behaviour was observed for the bandgap of SnS in which increasing the unit cell volume caused considerable bandgap widening, which was explained by considering the ECoN. We fabricated a highly oriented *Pnma*-type SnS <111> film without cogenerating any other Sn–S phases using gaseous H_2_S via RF-magnetron sputtering. By alloying with Ca, Sr, or Ba, whose ionic radii are larger than that of Sn^2+^, the bandgap of the SnS films could be widened beyond *E*_g_ = 2 eV while maintaining the strong <111**> **orientation. Cl as a doping agent was easily taken into the Ca-, Sr-, and Ba-alloyed SnS films and was solidified even at a high temperature of 580 °C. Time-dependent Seebeck measurements revealed that the conduction type of the Ca-alloyed SnS film could be converted from *p*- to *n*-type by reacting with liquidus SnCl_4_.

## Methods

### Theoretical prediction of the bandgaps of SnS (*Pnma*) and GeS (*Pnma*)

First-principles calculations of *Pnma*-type SnS and GeS were carried out with the projector augmented wave method^51^ implemented by the Vienna Ab-initio Simulation Package (VASP) code^52,53^. The cut-off energy was set to 600 eV, and the exchange-correlational term was evaluated with the generalised gradient approximation proposed by Perdew, Burke, and Ernzerhof (GGA-PBE)^54^. The initial electron configurations were 5*s*^2^ 5*p*^2^ 4*d*^10^ , 4*s*^2^ 4*p*^2^ 3*d*^10^ and 3*s*^2^ 3*p*^4^ for Sn, Ge and S, respectively. The sampling of *k*-points was performed using a Monkhorst–Pack 3 × 8 × 8 mesh^55^. The lattice parameters and internal atomic positions were fully relaxed until the residual stress and forces were below 0.1 GPa and 0.01 eV/Å, respectively. Convergence tests were carried out using more severe conditions, with 800 eV as the cut-off energy and a 6 × 16 × 16 k-mesh. Then, we confirmed that the total energy converged with 1 meV/atom.

### Deposition of SnS films and structural analysis

The SnS thin films were fabricated on quartz glass substrates (30 mm × 30 mm) using RF-magnetron sputtering. The instrument was equipped with three independent 1 in. cathodes, an introduction line for the gaseous H_2_S source, and a resistance substrate heater. The deposition time was 20 min for all experiments. The purity of the Ca target was 99.9% (Kojundo Chemical Laboratory Co., Ltd., Japan). The Sn, Sn–Cl (15 mol% SnCl_2_ mixed with Sn), and 1 in. disc Sr and Ba targets were made from metal reagents (Sn: 99.99%, Kojundo Chemical Laboratory Co., Ltd.; Sr: 99.99%, Sigma Aldrich; Ba: 99.99%, Sigma Aldrich) and formed using a pressing machine with a pressing maximum of 100 ton/cm^2^ in our laboratory. The SnS films were deposited at 320 °C with 1 sccm of H_2_S gas and 5 sccm of Ar working gas while an RF power of 14 W was applied to the metal Sn target. The total pressure was adjusted to 0.7 Pa. Before deposition, the sputtering chamber was baked before every deposition process and reached below the base pressure of 2 × 10^–5^ Pa. The A.E.-alloyed SnS films were deposited by applying RF power to the Ca, Sr, or Ba targets. The amount of each A.E. element in the films was adjusted by controlling the applied RF power. In the Cl doping experiments, the RF power applied to the metal Sn and SnCl_2_-mixed-Sn targets was adjusted to achieve the desired Cl content. The SnCl_2_ (15 mol.%)-mixed 1 in. Sn target (Sn–Cl target) was made in the laboratory using a pressing machine. The film thickness was nearly constant at 200 nm throughout the experiments (minimum of 160 nm, maximum of 230 nm).

The structural properties of the deposited films were analysed using XRD (RINT-2200, RIGAKU Co., Ltd., Japan) and Raman spectroscopy (inVia Raman microscope, excitation at 532 nm, RENISHAW Co., Ltd., UK). The optical properties were measured using a spectrophotometer (SHIMADZU, Solid Spec 3200 DUV, Japan). Seebeck measurements were carried out using a hot plate (IKA-KK, C-MAG HS4, Japan) and a digital multi-meter (KEYSIGHT Technologies, 34461A, USA).

## Supplementary Information

Supplementary Information.
