# Supplementary material for: Tunability of the bandgap of SnS by variation of the cell volume by alloying with A.E. elements
Source: Sci Rep. 2022 May 6;12:7434. doi: 10.1038/s41598-022-11074-2 (PMC9076691; doi:10.1038/s41598-022-11074-2)
Supplement: Supplementary file 1 — Supplementary Information. [file 41598_2022_11074_MOESM1_ESM.docx]

**Additional information**

**Supplementary information**


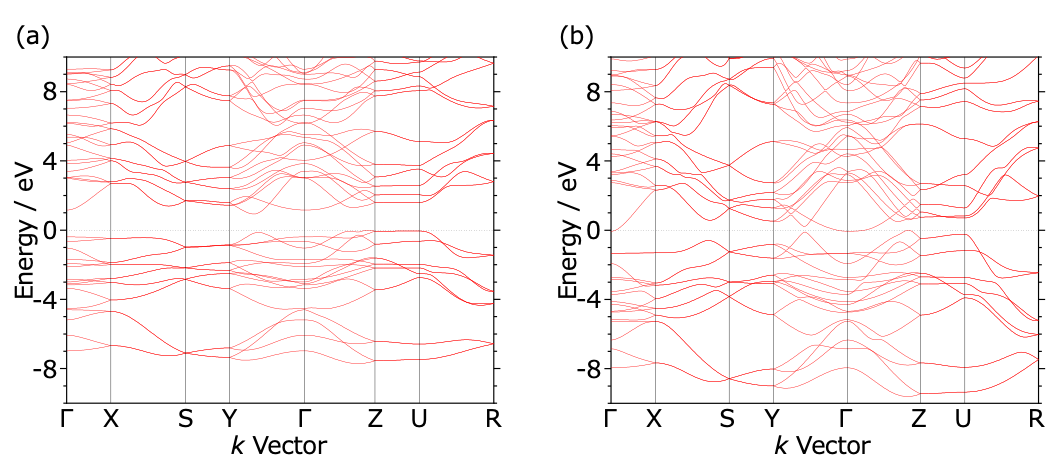


**Supplementary Figure 1.** Comparison between the band structures of SnS with unit cell volumes of a) 205.18 Å (corresponding to 0 GPa) and b) 169.06 Å (corresponding to 9 GPa).


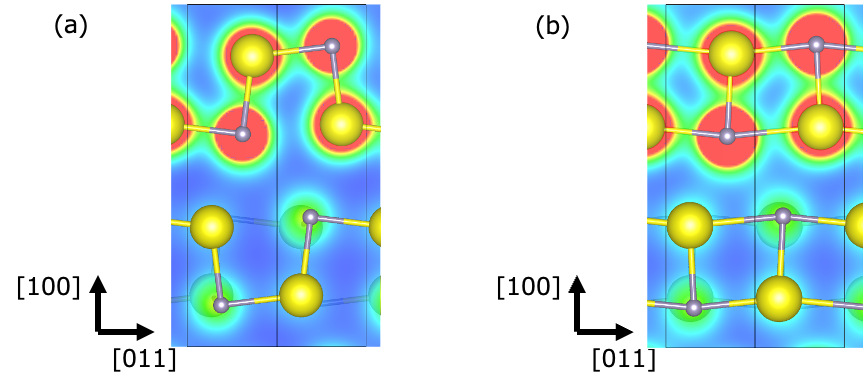


**Supplementary Figure 2.** Distribution of electron density in SnS with unit cell volumes of a) 205.18 Å (corresponding to 0 GPa) and b) 169.06 Å (corresponding to 9 GPa).

**Supplementary Figure 3.** XPS spectra which were measured using a hemispherical electron-energy analyzer (ULVAC-PHI.Inc:φ1800) with Mg Kα X-ray source (hν = 1253.6 eV).
